# Co-Cultivation—A Powerful Emerging Tool for Enhancing the Chemical Diversity of Microorganisms

**DOI:** 10.3390/md12021043

**Published:** 2014-02-17

**Authors:** Andreas Marmann, Amal H. Aly, Wenhan Lin, Bingui Wang, Peter Proksch

**Affiliations:** 1 Institute of Pharmaceutical Biology and Biotechnology, Heinrich-Heine University, Universitaetsstrasse 1, Bldg. 26.23, Duesseldorf 40225, Germany; E-Mails: andreas.marmann@uni-duesseldorf.de (A.M.); amal.hassan@uni-duesseldorf.de (A.H.A.); 2 State Key Laboratory of Natural and Biomimetic Drugs, Peking University, Health Science Center, Beijing 100191, China; E-Mail: whlin@bjmu.edu.cn; 3 Key Laboratory of Experimental Marine Biology, Institute of Oceanology, Chinese Academy of Sciences, Nanhai Road 7, Qingdao 266071, China; E-Mail: wangbg@ms.qdio.ac.cn

**Keywords:** marine-derived microorganisms, co-cultivation, mixed fermentation, silent genes, natural products

## Abstract

Marine-derived bacteria and fungi are promising sources of novel bioactive compounds that are important for drug discovery programs. However, as encountered in terrestrial microorganisms there is a high rate of redundancy that results in the frequent re-discovery of known compounds. Apparently only a part of the biosynthetic genes that are harbored by fungi and bacteria are transcribed under routine laboratory conditions which involve cultivation of axenic microbial strains. Many biosynthetic genes remain silent and are not expressed *in vitro* thereby seriously limiting the chemical diversity of microbial compounds that can be obtained through fermentation. In contrast to this, co-cultivation (also called mixed fermentation) of two or more different microorganisms tries to mimic the ecological situation where microorganisms always co-exist within complex microbial communities. The competition or antagonism experienced during co-cultivation is shown to lead to a significantly enhanced production of constitutively present compounds and/or to an accumulation of cryptic compounds that are not detected in axenic cultures of the producing strain. This review highlights the power of co-cultivation for increasing the chemical diversity of bacteria and fungi drawing on published studies from the marine and from the terrestrial habitat alike.

## 1. Introduction

Marine macroorganisms are already firmly established as sources of new drugs for the treatment of cancer but also for other malignancies such as chronic pain. So far, seven marine natural products or drugs derived from marine leads have entered the drug market. These compounds include among others Yondelis^®^ (approved for use in Europe, Russia and in South Korea for the treatment of soft tissue sarcoma), Adcetris^®^ (approved in the US and in the EU for the treatment of non-Hodgkin lymphoma or following an autologous stem cell transplantation), Halaven^®^ (approved for use in the US, Canada and in the EU for the treatment of advanced mamma carcinoma) and Prialt^®^ (approved in the US and in the EU for treatment of severe and chronic pain) [1,2,3,4,5,6,7,8,9]. In addition, the nucleoside analogues Cytosar-U^®^ and Vira-A^®^ may be mentioned that were modeled based on compounds isolated from marine sponges [7,10,11]. Many other compounds that are mainly derived from marine invertebrates are currently in clinical phases I or II, primarily for the treatment of cancer [12,13,14,15].

Research on marine-derived microorganisms is far more recent in comparison and was only seriously initiated in the nineties of the last century. As for marine macroorganisms, marine-derived bacteria and fungi quickly proved to be prolific sources of novel bioactive compounds that are of considerable interest as new drug leads. The most famous example of a marine microbial compound is salinosporamide A (marizomib), which is a fermentation product of marine *Salinispora* bacteria and a proteasome inhibitor that is currently undergoing clinical studies as a potential new anti-cancer drug [16,17,18,19]. It may be expected that further drug candidates obtained from marine-derived microorganisms will follow in the future.

However, as for natural products from terrestrial sources, the re-discovery of already known compounds is high and poses serious problems for marine bioprospecting from macroorganisms and microorganisms alike. New insights into the molecular biology of bacteria and fungi have demonstrated that the genetic potential of these microbes, in terms of producing a far greater chemical diversity of compounds than is currently known, has been vastly underestimated in the past [20,21,22]. Many microbial biosynthetic genes are apparently not transcribed under standard laboratory conditions but remain silent. As a consequence, only a fraction of the real biosynthetic diversity of microbes is obtained in terms of produced compounds, which leads to the currently experienced bottle neck in drug discovery from microbial sources. Several strategies exist that try to overcome these limitations during fermentation of microbes. These include among others the OSMAC approach where promising strains are cultured in a variety of media and under different culture regimes in order to maximize the diversity of compounds produced [23], in addition to epigenetic modifications. In the latter case microorganisms are treated with epigenetic modifiers such as histone deacetylase inhibitors or DNA methyl transferase inhibitors aiming at a modulation of histones or of the DNA thereby initiating the transcription of silent genes which in turn may lead to the accumulation of new compounds [24,25,26,27,28,29].

A third option tries to mimic the natural ecological situation, where microbes always co-exist within complex microbial communities. Competition for limited resources and antagonism are characteristics of these micro-habitats, which favor various defense mechanisms that rely mainly on the production of bioactive secondary metabolites [30]. It is often assumed that antibiotic production by bacteria and fungi can be interpreted within these ecological frames that will select for chemically defended microbes [31,32,33,34,35] even though other authors question this hypothesis [36,37,38]. Co-cultivation of two or more different microbes tries to mimic this setting in a laboratory scale. Competition among these microbes is deliberately provoked in the hope that biosynthetic genes that remain silent under luxurious culture conditions are activated and transcribed under stress conditions.

As shown in this review, several co-cultivation studies that have been conducted in recent years prove the feasibility of this strategy and suggest that co-cultivation is a viable experimental approach for enhancing the chemical diversity of microorganisms when grown *in vitro*. When preparing this review we have deliberately decided to extend our scope beyond co-cultivation studies reported from the marine field and to include those that have been carried out with microorganisms derived from the terrestrial habitat. Several reasons were decisive for this decision: first of all, the definition of a “marine” microorganism is often vague. Many microorganisms isolated from marine sources are in fact already known from the terrestrial habitat and can be cultured without the presence of sea salt in the medium. This is especially common for marine-derived fungi and has been noted by many authors [39,40,41]. It is in fact often assumed that a large part of the microbes found in the sea are of terrestrial origin and may survive under marine conditions but are by no means obligatory marine [42,43,44]. Furthermore, recent studies conducted with the fungus *Aspergillus nidulans* when challenged by *Streptomyces rapamycinicus* shed for the first time light on the molecular basis of induction of silent fungal biosynthetic gene clusters during co-cultivation [45,46,47]. These studies will certainly prove helpful for natural product chemists and molecular microbiologists who are trying to unravel the interspecies cross talk of marine-derived microorganisms in the future.

## 2. Results

### 2.1. Co-Cultivation Studies of Marine-Derived Microorganisms with Influence on Natural Product Accumulation

**Figure 1:**
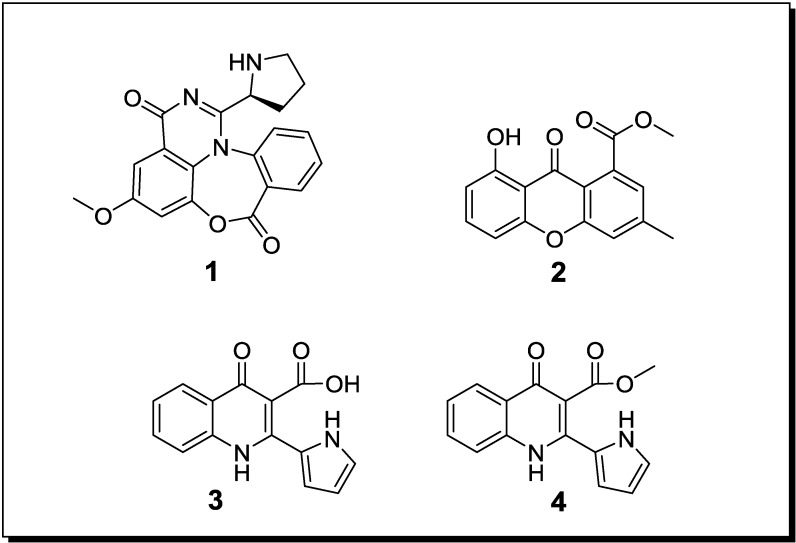
New natural products reported from co-cultures of marine-derived fungi.

**Figure 2:**
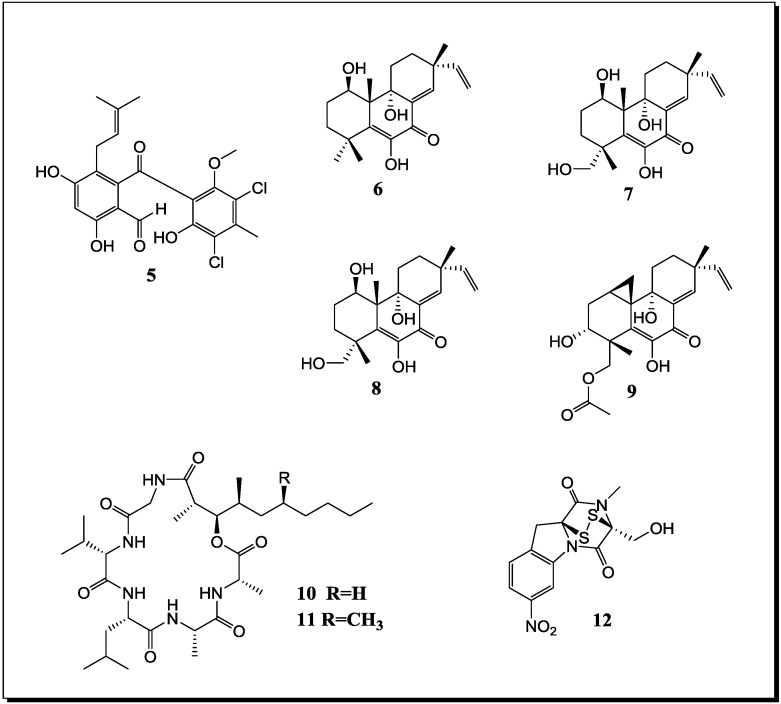
New natural products reported from co-cultures of marine-derived fungi and bacteria.

During the ongoing search for novel bioactive metabolites from microorganisms, several studies which were conducted mainly within the last few years proved the power of co-cultivation (also called “mixed fermentation”) as an experimental tool for either enhancing the production of constitutively present compounds and/or for inducing silent gene clusters. In general, the performed studies followed three major strategies which include (a) co-cultivation of different fungi; (b) co-cultivation of fungi with bacteria and (c) co-cultivation of different bacteria. With regard to marine-derived microorganisms (Table 1, Figure 1 and Figure 2 for structures) thirteen different co-cultivation studies have been published in recent years [34,48,49,50,51,52,53,54,55,56,57,58,59] proving that this experimental approach for increasing the chemical productivity of microbes is a highly promising strategy whereas on the other side it is still an emerging field that will require broader attention of marine natural product chemists and microbiologists alike in the future.

**Table 1:** Co-cultures of marine-derived microorganisms and secondary metabolites reported.

Co-Cultivated Microorganisms	Secondary Metabolites Reported	Reported Activity	Reference
*Aspergillus* sp. *Aspergillus* sp.	Aspergicin (n, **1**) Neoaspergillic acid (k) Ergosterol (k)	Antibiotic Antibiotic -	[48]
Unidentified fungus Unidentified fungus	8-Hydroxy-3-methyl-9-oxo-9*H*-xanthene-1-carboxylic acid methylether (n, **2**)	Antifungal	[49]
Unidentified fungus Unidentified fungus	Marinamide (n, **3**) Marinamide methylether (n, **4**)	Cytotoxic Cytotoxic	[60]
*Pestalotia* sp. *Unidentified bacterium*	Pestalone (n, **5**)	-	[52]
*Libertella* sp. *Thalassopia* sp.	Libertellenones A–D (n, **6**–**9**)	Cytotoxic	[53]
*Emericella* ap. *Salinospora arenicola*	Emericellamide A (n, **10**) Emericellamide B (n, **11**)	Antibiotic Cytotoxic	[54]
*Aspergillus fumigatus Sphingomonas* sp.	Glionitrin A (n, **12**)	Cytotoxic Antibiotic	[55]
*B. thuringensis* *B. Megaterium* *S. sciuri*	Indole (k) Phe-Pro diketopiperazine (k)	Antibiotic (on extract level)	[59]
*Streptomyces tenjimariensis* 12 unidentified bacteria	Istamycin (k)	Antibiotic	[34]

n = new; k = known.

In an attempt to induce the accumulation of novel compounds through fungal-fungal co-cultivation, Zhu *et al.* [48] demonstrated that the mixed cultivation of two different mangrove-derived epiphytic fungi, both belonging to the genus *Aspergillus*, leads to the production of the new alkaloid aspergicin (**1**) (Figure 1) and the known compounds neoaspergillic acid and ergosterol, respectively. Aspergicin (**1**) and neoaspergillic acid were evaluated for their antibiotic potential towards the Gram-positive bacteria *Staphylococcus aureus*, *Staphylococcus epidermidis*, *Bacillus subtilis*, *Bacillus dysenteriae*, *Bacillus proteus* and against the Gram-negative *Escherichia coli*. Aspergicin (**1**) exhibited a MIC of 15.62 µg/mL against *B. subtilis*, whereas the MIC of neoaspergillic acid was in the range of 0.49–15.62 µg/mL against all tested bacteria [48].

Li *et al*. [49] showed that the co-cultivation of two epiphytic, unidentified mangrove-associated fungi resulted in the production of a novel xanthone derivative (**2**). Using the agar plate diffusion assay and a fixed compound concentration of 100 µg/mL, a mild antifungal activity of **2** against *Gloeosporium musae* and *Peronophthora cichoralearum* was detected [49].

Co-cultivation of two unidentified mangrove-derived endophytic fungi, resulted in the production of the new alkaloids marinamide (**3**) and marinamide methylether (**4**) [50]. When studied for their cytotoxic effects on HepG2, 95-D, MGC832 and HeLa cells, IC_50_ values for marinamide (**3**) were in the nanomolar range whereas those of marinamide methylether (**4**) were in the low micromolar range [60].

Until now five studies were published on the co-cultivation of marine-derived fungi and bacteria. Miao *et al*. [51] investigated the influence of bacterial cells and cell free bacterial broth on the fungus *Arthrinium* c.f. *saccharicola*. In total the fungus was co-cultivated with 14 different fouling bacterial taxa. Six of them, including *Pseudoalteromonas spongiae*, *Shewanella algae*, *Vibrio vulnificus*, *Vibrio halioticoli*, *Pseudoalteromonas piscicida* and *Loktanella hongkongensis* reduced the growth of the fungus during co-cultivation. This effect was especially pronounced for *P. piscicida*. Addition of the cell free culture broth of this bacterium significantly increased the antimicrobial activity of the fungus when measured against *V. vulnificus* and *P. spongiae*. Apparently the induction of antibiotic activity by the fungus was caused by so far unknown bacterial compounds that had been secreted into the culture broth and served as chemical signals for elicitation of the fungal secondary metabolism [51].

In 2001 Cueto *et al.* [52] reported the chlorinated prenylsecoanthraquinone, pestalone (**5**) (Figure 2), from a co-culture of the marine-derived fungus *Pestalotia* sp. with a likewise marine-derived unidentified Gram-negative bacterium of the genus *Thalassopia* (CNJ-328). Since the methyl analogue of **5** was already known from another fungus (*Chrysosporium* sp.), pestalone (**5**) was suspected to be of fungal rather than of bacterial origin. When evaluating the antibacterial activity of **5 **against MRSA and against vancomycin-resistent *Enterococcus faecium*, MIC values of 37 ng/mL and 78 ng/mL were obtained whereas the GI_50_ of pestalone when measured in the NIH human tumor cell line screen amounted to 6.0 µM [52].

Co-cultivation of a marine-derived *Libertella* sp. fungus and the marine-derived bacterium *Thalassopia* sp. (CNJ-328) yielded the novel diterpenoids libertellenones A–D (**6**–**9**) which are presumably of fungal origin since diterpenes are far more common fungal rather than bacterial secondary products. The cell free bacterial culture broth as well as autoclaved cultures of CNJ-328 or an EtOAc extract of the bacterium failed to induce the accumulation of **6**–**9** by the fungus *Libertella* sp. Therefore a direct physical contact of fungus and bacterium is assumed to be necessary for induction of the libertellenones [53]. In spite of being induced by a bacterium, the libertellenones failed to show antibiotic properties against CNJ-328, MRSA or against a vancomycin-resistent *E. faecium* strain whereas they exhibited cytotoxic activity against HCT-116 cells with IC_50_ values between 0.76 and 53 µM, dependent on the derivative investigated [53]. Interestingly, in the last two studies the bacterial strain CNJ-328 was found to induce different biosynthetic pathways in *Libertella* sp. and in *Pestalotia* sp., respectively. Whereas the core structure of pestalone (**5**) is a polyketide which features a prenyl substituent, the libertellenones (**6**–**9**) are terpenoids.

Through co-cultivation of the fungus *Emericella* sp., that had been isolated from the surface of the marine green alga *Halimeda* sp. and the marine sediment-derived actinomyete *Salinispora arenicola* Oh *et al.* (2007) [54] were able to demonstrate a 100 fold increased production of the two fungal depsipetides emericellamide A (**10**) and B (**11**). Both compounds when evaluated for their biological activities displayed a moderate antibiotic activity against a methicillin-resistant *S. aureus* strain with MICs of 3.0 µM and 6.0 µM, as well as weak cytotoxic effects towards HCT-116 cells with IC_50_ values of 23 µM and 40 µM, respectively [54].

A novel diketopiperazine disulfide, glionitrin A (**12**), was obtained from a mixed fermentation of the marine-derived fungus *Aspergillus fumigatus* and the marine-derived bacterium *Sphingomonas* sp.. The compound showed strong cytotoxic activity against HCT-116, A549, AGS and DU145 cells with IC_50_ values of 0.82, 0.55, 0.45 and 0.24 µM, respectively, whereas the MIC against MRSA amounted to 0.78 µg/mL [55]. Interestingly, glionitrin A (**12**) exhibited also weak antifungal activity against *A. fumigatus*, which was used for the co-culture study. Presumably the core structure of glionitrin A (**12**) is derived from the fungus whereas the biogenetic origin of the nitro group is unknown [55].

Dusane *et al.* [56] isolated four surface associated bacteria from the green mussel *Perna viridis* and from the coral *Symphyllia* sp., which were identified as *Bacillus* sp., *Bacillus licheniformis*, *Bacillus pumilus* and as *Serratia marcescens* by analysis of their 16S rDNA sequences. Each bacterial strain was co-cultured with *Pseudomonas aeruginosa*, *Bacillus pumilus* and with the yeasts *Candida albicans* and *Yarrowia lipolytica.* The extracts resulting from co-cultivation were screened for increased antimicrobial activity against the respective competitors that had been used during mixed fermentation. Antibacterial activity against *P. aeruginosa* or against *B. pumilus* was enhanced for most of the studied epibiotic bacteria during co-cultivation. Biosurfactant production was increased when *B. pumilus* was co-cultivated with *S. marcescens* or with *B. lichenifomis*, whereas quorum-sensing inhibition was found for *Bacillus* sp. when co-cultured with *P. aeruginosa* [56].

In a similar approach Burgess *et al.* [57] isolated several unidentified surface associated bacteria form the marine algae *Fucus vesiculosus*, *F. serratus*, *Corallina officinalis*, *Ophiothrix fragilis* and *Asterias rubens*. In total 78 bacterial strains were isolated, cultured and systematically investigated for their antibiotic potential. Nine extracts showed antibiotic activity in the agar plate diffusion assay. Two unidentified strains, MH46 and SSE20, were chosen for a cultivation study employing cell free culture broths of *E. coli*, *B. subtilis* or of *P. aeruginosa* as well as of the unidentified surface associated bacterial strains MH1, MH2 and MH3. All resulting extracts were tested for their antibiotic properties against *B. subtilis*. Two major effects were noted: the antibiotically inactive bacterium MH46 suddenly showed antibiotic activity when exposed to the culture broth of *P. aeruginosa*. In addition the bacteria MH1, MH2 and MH3 and SSE20 showed an increased antibiotic activity when cultured in the presence of culture broths of MH1, MH2 and MH3 or of *B. subtilis* [57].

In a further study, sixteen strains of marine-derived epibiotic bacteria isolated from the algae *F. vesiculosus* and the nudibranch *Archidoris* *pseudoargus* were subjected to co-cultivation with the human pathogens *S. aureus*, *P. aeruginosa* and *E. coli* [58]. The marine-derived bacteria were cultivated either with autoclaved cultures of *S. aureus* or in the same culture vessels together with live cultures of the pathogens. In the latter case the pathogens were kept within dialysis tubes with a pore size of 8000 Da thereby allowing diffusion of secreted low to medium weight compounds but avoiding physical cell-cell contact of pathogens and marine-derived bacteria. As a read out, the antibiotic activity of the resulting extracts was measured using MSSA, MRSA, *E. coli* and *P. aeruginosa* as indicator strains. Both *S. aureus* and *P. aeruginosa* induced antibiotic activity in several of the marine-derived bacteria. This induced antibiotic activity was similar for live and for autoclaved bacteria [58].

When two different *Bacillus* spp. (related to *B. thuringiensis* and *B. megaterium,* respectively based on 16S rDNA sequence similarity) that had been isolated from the surface of the marine alga *Ulva californica* were co-cultured, the production of indole and (Phe-Pro) diketopiperazines was found to increase by 450% and 320%, respectively compared to an axenic culture of the producing strain (“*B. thuringiensis*”) [59]. This induced accumulation of diketopiperazines coincided with an increase of antibiotic activity against the second *Bacillus* strain (“*B. megaterium*”). When “*B. thuringiensis*” was co-cultured with another bacterial strain isolated from *U. californica* (*Staphylococcus sciuri*), no induction of diketopiperazine accumulation was detected. At the same time the analyzed indole and (Phe-Pro) diketopiperazines failed to exert antibiotic activity against *S. sciuri* [59].

In a further study Slattery *et al.* [34] investigated the interaction between the marine-derived *Streptomyces tenjimariensis* and 53 further unknown marine bacteria, with regard to induction of istamycin A and B accumulation by *S. tenjimariensis*. Twelve out of 53 co-cultures showed at least a twofold higher production of istamycins compared to an axenic culture of *S. tenjimariensis*. This effect was only found when *S. tenjimariensis* was inoculated 24 h prior to the other bacteria. When the bacterial competitors had been inoculated 24 h before addition of *S. tenjimariensis*, or when both cultures were inoculated simultaneously a reduced istamycin production was observed [34].

### 2.2. Co-Cultivation Studies of Terrestrial Microorganisms with Influence on Natural Product Accumulation

**Figure 3:**
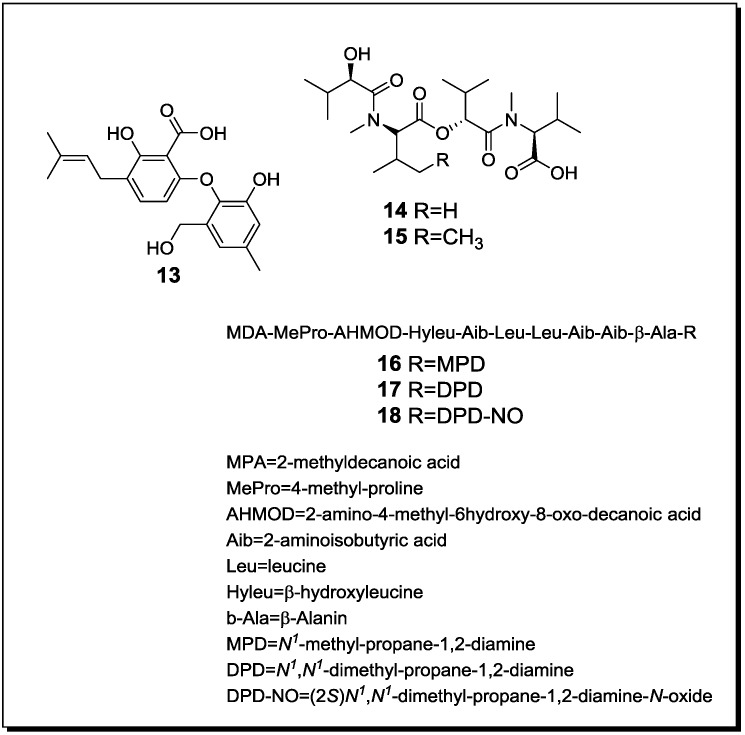
New natural products reported from co-cultures of terrestrial fungi.

**Figure 4:**
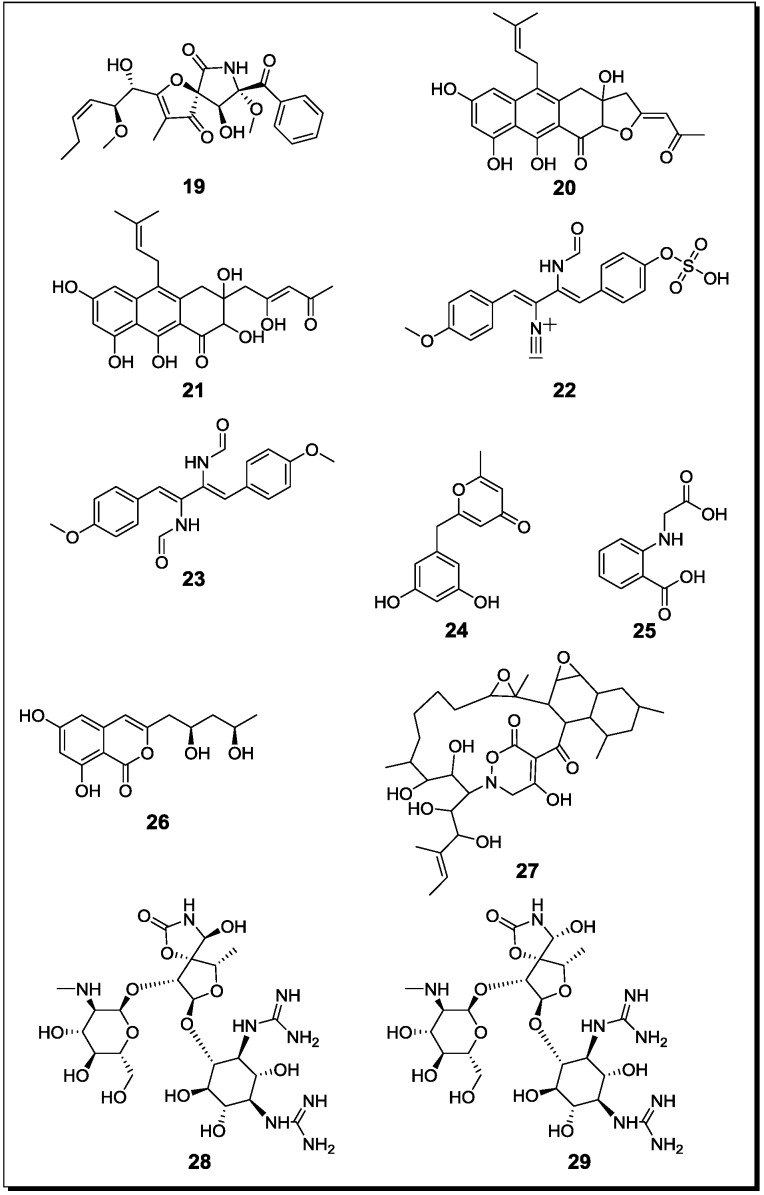
New natural products reported from co-cultures of terrestrial fungi with bacteria or from bacterial co-cultures.

Reported co-cultivation studies of terrestrial microorganisms follow similar research strategies as described before for marine-derived microbes and focus on interactions of fungi with fungi, fungi with bacteria or bacteria with bacteria. To the best of our knowledge seven studies investigating the effects of fungal-fungal interaction have been reported to date from the terrestrial habitat (Table 2, Figure 3 and Figure 4 for structures).

**Table 2:** Co-cultures of terrestrial microorganisms and secondary metabolites reported.

Co-Cultivated Microorganisms	Secondary Metabolites Reported	Reported Activity	Reference
*Penicillium pinophilum* *Trichoderma harzianum*	Secopenicillide C (n, **13**)	n.t.	[61]
	Penicillide (k)	n.t.	
	MC-141 (k)	n.t.	
	Pestalasin A (k)	n.t.	
	Stromemycin (k)	n.t.	
*Fusarium tricinctum* *Fusarium begoniae*	Subenniatin A (n, **14**)	-	[62]
	Subenniatin B (n, **15**)	-	
	Enniatin A (k)	-	
	Enniatin A_1_ (k)	-	
	Enniatin B (k)	-	
	Enniatin B_1_ (k)	-	
*Acremonium* sp. *Mycogone rosea*	Acremostatin A (n, **16**)	n.t.	[63]
	Acremostatin B (n, **17**)	n.t.	
	Acremostatin C (n, **18**)	n.t.	
*Gloeophyllum abietinum* *Heterobasidion annosum* *Armillaria ostoyae*	Oospoglycol (k)	n.t.	[64]
	Oopsonol (k)	n.t.	
	Fomannoxin (k)	n.t.	
	Fomannoxinalcohol (k)	n.t.	
	Fomannosin (k)	n.t.	
	Melledonal (k)	n.t.	
	Melledonal C (k)	n.t.	
	Melleolide D (k)	n.t.	
*Oyadendron sulphureoochraceum* *Ascochyta pisi* *Emercillopsis minima* *Cylindrocarpon destructans* *Fusarium oxysporum*	Lateritin (k)	Cytotoxic Antifungal Antibiotic	[65]
*Paraconiothyrium* sp. *Alternaria* sp. *Phomopsis* sp.	Paclitaxel (k)	n.t.	[66]
*Streptomyces bullii* *Aspergillus fumigatus*	Brevianamide F (k)	-/-/C	[67]
	Spirotryprostatin A (k)	T/L/C	
	6-Methoxy spirotrypostatin B (k)	-/L/C	
	Fumitremorgin C (k)	T/L/C	
	12,13-Dihydroxy Fumitremorgin C (k)	T/L/C	
	Fumitremorgin B (k)	T/L/C	
	Verruculogen (k)	T/L/C	
	11-*O*-Methylpseurotin A (k)	-/-/C	
	11-*O*-Methylpseurotin A*2* (n, *19*)	-/L/C	
	Ergosterol (k)	-/-/-	
	Emestrin A (k)	n.t.	
	Emestrin B (k)	n.t.	
*Aspergillus fumigatus* *Streptomyces rapamycinicus*	Fumicycline A (n, 20)	Antibiotic	[45]
	Fumicyline B (n, 21)	Antibiotic	
*Aspergillus fumigatus* *Streptomyces peucetius*	Fumiformamide (n, 22) *NN*′-((1*Z*,3*Z*)-1,4-bis (4-Methoxyphenyl)buta-1,3-diene-2,3diyl)Di-formamide (n, 23)	Cytotoxic	[68]
*Fusarium tricinctum* *Bacillus subtilis*	Macrocarpon C (n, 24)	-	[30]
	2-(Carboxymethylamino)benzoic acid (n, 25)	-	
	(−)-Citreoisocoumarinol (n, 26)	-	
	Lateropyrone (k)	Antibiotic	
	Enniatin A1 (k)	Antibiotic	
	Enniatin B (k)	-	
	Enniatin B1 (k)	Antibiotic	
	(+)-Citreoisocoumarinol (k)	-	
*Tsukamurella pulmonis* *Streptomyces endus*	Alchivemycin A (n, 27)	Antibiotic	[69]

n = new; k = known; n.t. = not tested; T = trypanocidal; L = leishmanocidial; C = cytotoxic; - = inactive.

Co-cultivation of the soil-derived fungi *Penicillium pinophilum* and *Trichoderma harzianum* [61] resulted in an enhanced production of penicillide, MC-141, stromemycin and pestalasin A and in an induction of the new natural product secopenicillide C (**13**) (Figure 3). All respective compounds were produced by *P. pinophilum* [61].

Co-cultivation of the plant endophytic fungi *Fusarium tricinctum* and *Fusarium begoniae* yielded the new linear depsipetides subenniatin A (**14**) and B (**15**), together with the known cyclic depsipeptides enniatins A, A_1_, B and B_1_ that had been previously reported from *F. tricinctum* [62]. In contrast to the cyclic enniatins, the new compounds failed to show activity against the murine lymphoma cell line L5178Y or against the bacterial pathogens *E. coli*, *S. aureus* and *P. aeruginosa*. It may be speculated that compounds **14** and **15 **are detoxification products of the cyclic enniatins that result from hydrolytic activity of *F. begoniae*. Interestingly, co-cultures of *F. tricinctum* and *Fusarium equiseti* failed to elicit the production of the new compounds **14 **and **15** [62].

When the fungus *Acremonium* sp. that had been obtained as an endophyte from *Taxus baccata* was challenged with the mycoparasite *Mycogone rosea*, the novel lipoaminopeptides acremostatins A (**16**), B (**17**) and C (**18**) were obtained [63]. By mass spectrometry, the new metabolites were found to be structurally related to the known leucinostatins A, B and K, but differed from the latter by the presence of 2-methyldecanoic acid instead of (4*S*,2*E*)-4-methylhex-2-enoic acid as terminal substituent [63].

When pair-wise cultivating the wood-derived fungi *Heterobasidion annosum*, *Gloeophyllum abietinum* and *Armillaria ostoyae*, an induction of antibacterial compounds and mycotoxins was observed [64]. While co-culturing *G. abietinum* with *H. annosum*, the growth of *G. abietinum* was reduced, but the production of the antibiotics oospogylcol and oosponol derived from *G. abietinum* was increased (approximately 40 fold in liquid culture and 250 fold on agar plates). Simultaneously, *H. annosum* was found to react to the co-culture conditions by an approximately 430 fold increased production of the toxins fomajorin S and fomannosin. When *A. ostoyae* was cultivated with *G. abietinum* or with *H. annosum*, respectively, similar results with regard to an induction of secondary products were obtained. It was hypothesized, that small molecules are responsible for inducing the secondary metabolism in the investigated fungi. To prove this hypothesis cell free medium, heat-sterilized fungal cultures, or chemically and mechanically disrupted fungal cell walls, as well as either low or high molecular weight compounds from *H. annosum* or from *G. abietinum* were studied for their ability to induce the secondary metabolism of *G. abietinum* and of *A. ostoyae*, respectively. It was shown that low molecular molecules from *H. annosum* of less than 3000 Da are responsible for the observed increased production of secondary metabolites by *G. abietinum.* It was furthermore suggested that this induced accumulation of secondary products is due to a *de novo* biosynthesis of the respective molecules, since addition of 10 µg/mL of cycloheximide to the co-cultures stopped oospoglycol accumulation [64].

Mixed fermentation of five sediment-derived fungi, including *Oyadendron sulphureoochraceum*, *Ascochyta pisi*, *Emercillopsis minima*, *Cylindrocarpon destructans* and *Fusarium oxysporum* yielded lateritin as a major metabolite [65]. Lateritin showed cytotoxic effects on P388, PXPC-3, MCF-7, CNS SF268, NSC H460, KM20L2 and DU-145 tumor cells with IC_50_ values ranging from 1.7 to 2.0 µM. In addition, the compound showed antimicrobial activity against *C. albicans*, *Micrococcus luteus*, *S. aureus*, *E. faecalis* and against *Streptococcus pneumoniae* with MIC values between 2 and 16 µg/mL [65].

A systematic investigation of paclitaxel production by the endophytic fungus *Paraconiothyrium* sp., derived from the wood of *Taxus x media*, indicated that co-cultivation of *Paraconiothyrium* sp. with other endophytic fungi such as *Alternaria* sp. and *Phomopsis* sp. enhanced paclitaxel production between 2.7 and 3.8 fold compared to axenically grown *Paraconiothyrium* sp. [66]. When all three fungi were cultivated together paclitaxel production was even increased 7.8 fold, suggesting a cumulative effect.

A change of the morphology and pigmentation of the fungus *Monascus* sp. was observed upon co-cultivation with *Saccharomyces cerevisae* or with *Aspergillus oryzae*, while co-cultivation with *Bacillus cereus* did not cause such an effect [70]. It was shown that the culture broth and the ammonium sulphate precipitate of the culture broth of *S. cerevisae* elicited similar effects with regard to growth and pigment production by *Monascus* sp., while the heat sterilized culture of *S. cerevisiae* did not. When investigating the enzyme activities of the culture broth of *S. cerevisae*, chitinase and amylase activity were found. The purified chitinase from *S. cerevisae* was observed to cause the previously observed morphological changes of *Monascus* sp., while the related enzymes lysozyme, α-amylase, chitinase and protease purified from *Bacillus* sp., *Streptomyces* sp. and *Staphylococcus* sp. had no effect. The morphological change of *Monascus* sp. was linked to the hydrolytic effect of the chitinase, whereas pigment production was assumed to be a defensive reaction aimed at inhibiting chitinase activity [70].

With regard to fungal-bacterial interactions, co-cultures of *Aspergillus fumigatus* and different *Streptomyces* sp. were investigated in more detail. Addition of the soil derived *Streptomyces bullii* from the Atacama Desert to a culture of *Aspergillus fumigatus* afforded ten compounds, including the diketopiperazines brevianamide F, spirotryprostatin A, 6-methoxy spirotryprostatin B, fumitremorgin C, 12,13-dihydroxy fumitremorgin C, fumitremorgin B, verruculogen, and the pseurotins 11-*O*-methylpseurotin A and its new isomer 11-*O*-methylpseurotin A_2_ (**19**) (Figure 4), as well as ergosterol [67]. None of these compounds were detected in axenic cultures of *A. fumigatus*. Interestingly, when assayed for their antibiotic activity against *S. aureus* and *E. coli*, none of the induced fungal compounds was found to be active. When the fungus was treated either with the cell-free bacterial culture broth of *S. bullii*, a methanolic extract derived from the bacterial culture or an autoclaved bacterial culture, no induction of fungal metabolites was observed. Addition of *N*-butyryl-dl-homoserin, a known bacterial quorum sensing inhibitor, to the fungal culture afforded the fungal metabolites emestrin A and B. All metabolites were evaluated for their trypanocidal, leishmanicidal and cytotoxic activity (the latter using MRC5 cells). Fumitremorgin C, 12,13-dihydroxy fumitremorgin C, fumitremorgin B and verruculogen showed high activities in the used test systems with MIC values in the low to medium micromolar range [67].

König *et al*. [45] demonstrated the production of the new fungal derived polyketides fumicyclines A (**20**) and B (**21**) in a mixed fermentation of *A. fumigatus* and *Streptomyces rapamycinicus*. Both compounds showed a weak antibiotic effect in the agar plate diffusion assay against *S. rapamycinicus*. When incubating the fungus with the cell free bacterial culture broth or when the fungus was co-cultivated with the bacterium kept in a dialysis tube, no induction of fumicyclines was observed, indicating that a direct physical contact of fungus and bacterium is necessary for elicitation of fungal metabolites. Electron microscopy of co-cultured fungus and bacterium revealed an intimate physical contact between the fungal mycelia and the bacterial filaments. It was concluded that *S. rapamycinicus* is able to alter gene expression in *A. fumigatus* by modulating regulatory processes. Treatment of the fungus with the known histone deacetylase inhibitor suberoylanilide hydroxamic acid (SAHA) caused an increased transcription level of the PKS gene that is involved in the biosynthesis of fumicyclines, while no PKS gene transcription was observed following treatment with the histone acetyltransferase inhibitor anacardic acid [45].

A further class of fungal compounds, xanthocillin analogues, was produced by *A. fumigatus* in the presence of *Streptomyces peucetius* [68]. The induced compounds included the new derivatives fumiformamide (**22**) and *N,N*′-((1*Z*,3*Z*)-1,4-bis(4-methoxyphenyl)buta-1,3-diene-2,3diyl)di-formamide (**23**), together with the known *N*-formyl derivatives and BU-4704. Compound **23** exhibited strong cytotoxic effects in the NCI-60 cell line screen with MIC values between 0.65 and 1.12 µM [68].

A co-culture of the fungal endophyte *F. tricinctum* and *B. subtilis*, resulted in an up to 78 fold enhanced production of the constitutively present fungal secondary metabolites lateropyrone, fusaristatin and enniatins A_1_, B and B_1_ compared to an axenic culture of *F. tricinctum* [30]. In addition, four compounds that included (+)-citreoisocoumarinol, the new macrocarpon C (**24**), 2-(carboxymethylamino)benzoic acid (**25**) and (−)-citreoisocoumarinol (**26**) were detected, which were absent in axenic cultures of both, the fungus and the bacterium. Interestingly, this induction of fungal metabolism was only found when *B. subtilis* was inoculated several days prior to the fungus to the culture medium. When inoculated simultaneously, the fungus inhibited the bacterium and no clear induction of fungal compounds was detected. When studied for their antibacterial properties, enniatins B_1_ and A_1_ showed MIC values against *B. subtilis* of 8 and 16 µg/mL, respectively, whereas the MIC values against the human pathogens *S. aureus*, *S. pneumoniae* and *E. faecalis* were between 2 and 8 µg/mL. Additionally, the constitutively present fungal metabolite lateropyrone exhibited MIC values between 2 and 8 µg/mL against all mentioned bacterial strains [30]. Interestingly, when co-cultured with two streptomycetes (*S. coelicolor* and *S. lividans*), *F. tricinctum* was shown to produce further, so far unknown products that differ from those detected during co-cultivation of the fungus and *B. subtilis*. This suggests specific reactions of the fungus in response to different bacteria.

When the yeast *C. albicans* was co-cultured with the bacterium *P. aeruginosa*, the formation of a pyocyanin related red pigment that was located intracellularly within *C. albicans* was observed [71]. It was suggested that a possible intermediate, 5-methyl-phenazinium-1-carboxylat (5MPCA) produced by *P. aeruginosa*, was transformed to the red pigment within the yeast. This hypothesis was corroborated by a feeding experiment with 5MPCA, which was shown to be transformed to the red pigment by the yeast. Co-cultivation of *C. albicans* with related bacterial taxa such as *Pseudomonas* sp., *P. putida*, *P. fluorescens* or *P. chlororaphis* failed to elicit the production of the red pigment, suggesting that pigment formation is a specific response of *C. albicans* to *P. aeruginosa*. Pigment formation was observed to coincide with a reduced fungal viability compared to controls [71].

Mycolic acid producing bacteria were shown to be able to elicit the accumulation of cryptic natural products in other bacteria. A mixed fermentation of *S. lividans* with the mycolic acid producing bacterium *Tsukamurella pulmonis* induced the production of a red pigment in *S. lividans* [69]. For pigment production, a direct cell-cell contact between *S. lividans* and *T. pulmonis* was found to be necessary. In the same study, *Streptomyces endus* was found to produce the new antibiotic alchivemycin A (**27**) when co-cultured with *T. pulmonis*. Alchivemycin A revealed a MIC of 0.06 µg/mL against *Micrococcus luteus* that was used as an indicator strain to assess antibiotic activity [69].

When investigating bacterial-bacterial interactions, the survival of *Salmonella duesseldorf* in soil in the presence of *Streptomyces lividans* or *Streptomyces bikiniensis* was investigated [72]. While *S. bikiniensis* is a known streptomycin producer, *S. lividans* is a non-antibiotic producer. *S. duesseldorf* was shown to be sensitive towards streptomycin, resulting in a reduced survival of *S. duesseldorf* in the presence of *S. bikiniensis* in non-sterile and sterile amended soil. Competition in sterile amended soil indicated a greater survival of *S. duesseldorf* in the presence of *S. lividans* compared to axenically grown *S. duesseldorf*. It was hypothesized that the presence of vegetative mycelia of *S. lividans* has a positive effect on the accessibility of nutrients by *S. duesseldorf.* The study provided evidence for antibiotic production in soil with regard to accumulation of streptomycin by *S. bikiniensis* [72].

With the purpose of investigating the role of antibiotics [31], two *Streptomyces* spp. were co-cultured with *B. subtilis* under semi-ecological conditions. The *Streptomyces* spp. used for this study included *S. griseus* and *S. coelicolor.* Three different experimental set ups were investigated: *B. subtilis* was first established in a growth medium which was then consecutively inoculated with one of the two *Streptomyces* spp. Alternatively, *B. subtilis* and a chosen *Streptomyces* strain were inoculated simultaneously, or as a third alternative *Streptomyces* sp. was inoculated prior to addition of *B. subtilis*. Antibiotic production by *S. griseus* and by *S. coelicolor* was found to prevent invasion by *B. subtilis*, whereas no advantage for the streptomycetes was seen when invading an already existing population of *B. subtilis* [31].

### 2.3. Molecular Biology and Accumulation of Cryptic Natural Products in Mixed Fermentations of the Model Organism Aspergillus Nidulans and Streptomyces Rapamycinicus

Since the genome of *Aspergillus nidulans* is known from sequencing, this fungal species was chosen by the groups of Hertweck and Brackhage for detailed molecular studies related to the induced accumulation of natural products in mixed fermentations with soil-derived actinomycetes [46,47]. Bioinformatic genome analysis of *A. nidulans* revealed 28 putative polyketide and 24 putative nonribosomal peptide biosynthetic gene loci, giving the fungus theoretically the possibility to produce at least 52 different secondary metabolites [47]. Analysis of the secondary metabolites hitherto reported from *A. nidulans* when grown under standard laboratory conditions, however, indicated that only a small part of the biosynthetic genes are expressed. Using the *A. nidulans* secondary metabolism array (ASMA) which monitors the central enzymes of each biosynthetic pathway, the fungus was co-cultured with 58 different actinomycetes [46]. Fungal mRNA and cDNA were isolated during co-cultivation and hybridized with the ASMA. Interestingly, only one actinomycete strain, *Streptomyces rapamycinicus*, led to an up-regulation of two putative fungal PKS and NRPS genes, AN7909 and AN7884. Using full-genome microarrays, 395 genes were found to be differentially expressed in *A. nidulans* when co-cultured with *S. rapamycinicus,* including 248 genes that were up-regulated, while 147 genes were down-regulated in the region between AN7874 and AN7914. In order to determine the gene regulating mechanism present in *S. rapamycinicus*, *A. nidulans* was monitored by qRT-PCR, while being incubated either with the cell-free bacterial broth, with (co)culture-derived extracts, with heat killed bacteria or with living bacteria that were kept in a dialysis tube and were thus physically separated from the fungus. None of these experiments led to the activation of fungal gene clusters, indicating the necessity of an intimate contact between fungus and streptomycete for fungal gene modulation. Electron microscopy showed a close physical interaction of both microorganisms. HPLC analysis of axenic fungal and bacterial cultures and of mixed-fermentations of *A. nidulans* and *S. rapamycinicus* demonstrated striking differences. Mixed fermentations of *A. nidulans* and *S*. *rapamycinicus* yielded four cryptic fungal compounds that included orsellinic acid, lecanoric acid, F-9775 and F-9775B. None of these metabolites were detected either in axenic cultures of the fungus or in PKS lacking mutants of *A. nidulans* [46].

In order to investigate the mechanism of gene regulation in *A. nidulans* in more detail, the fungus was treated with the histone acetyltransferase inhibitor anacardic acid or with the histone deacetylase inhibitor suberoylanilide hydroxamic acid (SAHA) [47]. The histone deacetylase inhibitor SAHA caused an activation of fungal gene expression, as observed during co-cultivation of *A. nidulans* with *S. rapamycinicus*, indicating that also the streptomycete triggers modification of fungal histones. Treatment with anacardic acid inhibited fungal biosynthetic gene expression. Upon deleting 36 out of 40 fungal histone acetyltransferases, it was demonstrated that the fungal Saga/Ada complex that contains the histone acetyltransferase GcnE and the AdaB protein is required for induction of the fungal orsellinic acid gene cluster by *S. rapamycinicus*. A Saga/Ada dependent increase of histone 3 acetylation at the lysine residue 9 was found to take place during co-cultivation of the fungus and the streptomycete proving for the first time that fungal histone acetylation is caused by interaction with the streptomycete, and that this increased histone acetylation in turn leads to an accumulation of cryptic fungal natural products [47].

## 3. Discussion and Outlook

The studies reported in this review even though still limited in number clearly underline the power of co-cultivation as an emerging tool to increase the chemical diversity of secondary products that are produced by fungi or bacteria during *in vitro* fermentation. Co-cultivation is expected in the future to routinely complement other experimental approaches that likewise aim at diversifying secondary compound production by microorganisms such as mutagenesis [23,47], the OSMAC approach [23] or treatment of microbes with epigenetic modifiers [24] to name just a few. In contrast to the latter methods, co-cultivation is an ecologically driven approach that tries to mimic the natural situation where a given microbe is always embedded in a more or less complex microbial community and exposed to a multitude of chemical signals that are exchanged between the different taxa that compose this community. This chemical interaction will be even more complex if one considers that many marine-derived microbes are not free living in seawater or in the sediment, but are members of a microbial community that are e.g., hosted by marine invertebrates [73,74,75,76,77] or within algae [78,79,80,81] or even mangroves [82,83,84,85]. Compounds produced by the hosts are likely to have an influence on this microbial community too and may even act as stimulating clues that induce the production of certain microbial compounds that may not be accumulated in the absence of these clues.

Even when considering only the microbial inter-species crosstalk as discussed in this review, the scenario is complex and far from being understood. This is especially true for the mostly unknown nature of chemical triggers or signals that lead to an accumulation of cryptic natural products that are not detected in axenic microbial cultures and/or to a significantly enhanced production of constitutively present compounds. Several reports that have been highlighted in this review indicate specific reactions of fungi or bacteria during co-cultivation experiments that involve more than one microbial partner. For example, co-cultivation of two Bacillus strains that resembled *B. thuringiensis* and *B. megaterium* caused an increased production of indole and (Phe-Pro) diketopiperazines by the producing strain (“*B. thuringiensis*”), whereas co-cultivation of the latter with another marine-derived bacterial strain had no influence on diketopiperazine accumulation [59]. Interestingly, the induction of diketopiperazine production coincides with the susceptibility of inducing *vs*. non-inducing bacterial strains towards these secondary compounds [59]. Two further examples come from the terrestrial habitat and involve the cross talk between a fungus and different bacteria. Out of 58 different actinomycetes that were co-cultured with the fungus *A. nidulans*, only *S. rapamycinicus* was able to induce the accumulation of orsellinic acid and of biogenetically related compounds in the fungus [46]. When the fungal endophyte *F. tricinctum* was co-cultured with *B. subtilis*, the induced fungal natural products differed from those that were detected during co-cultivation of the fungus with either *Streptomyces coelicolor* or with *S. lividans* [30]. These different metabolic reactions of either bacteria or fungi during co-cultivation with a small set of other microbes argue for the presence of different, perhaps even specific clues or signal molecules that can apparently be perceived by the producing microbial strain. Whereas it is relatively easy to observe the metabolic reactions of fungi or bacteria when exposed to these different clues, it is far more difficult and challenging to identify the signals that are responsible for inducing the accumulation of cryptic natural products. Several studies have tried to locate these signals e.g., in the cell free culture broth or in the cellular biomass of inducing microbial strains. For example, it has been shown that an induced production of libertellenones by the marine-derived fungus *Libertella* sp. depends on a close physical contact between the fungus and the inducing bacterium CNJ-328 [53]. On the other side, the study by Sonnenbichler *et al.* [64] demonstrated that the induced accumulation of the antibiotics oospogylcol and oosponol by the fungus *G. abietinum* was triggered by low molecular weight compounds of less than 3000 Da that are secreted into the culture broth by the inducing fungus *H. annosum*. On the contrary, accumulation of fumicyclines A and B by *A. nidulans* as induced by *S. rapamycinicus* was found to require a direct physical contact between the fungus and the streptomycete [45].

The induction of cryptic natural products or the significant enhancement of constitutively present secondary compounds is usually considered to be a stress response caused by the presence of a competing microorganism. Hence, induction of metabolites as a result of stress during co-cultivation should be part of the chemical defense of the producer and aimed at inhibiting or even killing the competitor. Rather surprisingly, this hypothesis has so far rarely been proven in co-cultivation studies. One notable example is the co-cultivation between the fungal endophyte *F. tricinctum* and *B. subtilis*. The fungal derived enniatin derivatives that were greatly enhanced during co-cultivation showed a clear antibiotic activity that extended not only to clinically relevant bacterial pathogens such as MRSA but also to *B. subtilis* that had been used as an inducer in the respective co-cultivation study [30]. Other studies failed to demonstrate such a protective effect. For example, in spite of being induced by the marine-derived bacterium CNJ-328, the libertellenones that were produced by the marine-derived fungus *Libertella* sp. showed no antibiotic activity against the inducing bacterium [53].

The cross talk between inducer and producer during co-cultivation of microorganisms is a highly dynamic process that is also influenced by time. The enhanced production of istamycins by the marine-derived *S. tenjimariensis* during co-cultivation with several unknown likewise marine-derived bacteria was only detected when the istamycin producer was inoculated 24 h prior to the inducers into the culture broth. No enhancement of istamycin production was found when producer and inducer were inoculated at the same time [34]. The demonstration of the time dependent istamycin production [34] gives further evidence for the rat race between the microorganisms within a microbial community. Time was also shown to be an important factor during co-cultivation of the fungal endophyte *F. tricinctum* and *B. subtilis*. When inoculated at the same time, the fungus clearly out competed the bacterium and no clear changes in the fungal metabolite profile were visible. When the bacterium was given a head start during cultivation, the growth of the fungus was slowed down and a clear induction of fungal metabolites occurred [30].

One further aspect which is horizontal gene transfer and which may perhaps also influence the result of co-cultivation studies should finally not be overlooked. During co-cultivation of a multi-antibiotic resistant strain of the bacterium *Rhodococcus fascians* that by itself does not produce antibiotics and a strain of *Streptomyces padanus* that is an actinomycin producer a strain of *Rhodococcus* emerged that was found to contain large segments of *Streptomyces* DNA and produced the new aminoglycosides rhodostreptomycins A (**28**) and B (**29**) [86]. These compounds differ considerably from the actinomycins that are produced by *S. padanus* and have been interpreted to arise from horizontal gene transfer of the streptomycete to *Rhodococcus* during co-cultivation.

For the fungus *A. nidulans*, for which the genome is known through sequencing and which can therefore be considered to be a model organism, the molecular basis underlying the induction of orsellinic acid derived secondary metabolites and of other compounds by *S. rapamycinicus* could be demonstrated for the first time [46]. The interaction of fungus and streptomycete leads to an increased histone acetylation within gene clusters encoding for fungal natural products. This in turn leads to an induced accumulation of cryptic compounds that are not detected when the fungus is grown under axenic conditions. Interestingly an induced accumulation of fungal products was also observed when the fungus was treated with the HDAC inhibitor SAHA [47] that is also used as a tool in epigenetic studies that aim likewise at an induction of cryptic biogenetic pathways [24] thereby providing a link between co-cultivation and epigenetic modulation as two emerging tools that aim jointly at enhancing the chemical diversity of microorganisms.

It is clear that co-cultivation as an experimental tool, which aims at a diversified production of bioactive compounds that could be leads for biomedical research, is promising but nevertheless still in its infancy. Far more questions rather than answers or established protocols exist. Generalizations are dangerous, each case needs to be considered individually before any wider conclusions can be drawn. Nevertheless, taking this ecologically driven approach at enhancing the chemical diversity of microbes beyond the boundaries that can be reached by routine axenic cultivation is both thrilling and promising. It is hoped that far more researchers will adopt this approach for their own work in the future.
